# *Carthamus tinctorius* L.: a comprehensive review of its ethnomedicine, phytochemistry, pharmacology, and clinical applications

**DOI:** 10.3389/fphar.2025.1609299

**Published:** 2025-08-21

**Authors:** Haotian Bai, Jing Yang, Rui Wang

**Affiliations:** ^1^ College of Pharmacy, Heilongjiang University of Chinese Medicine, Harbin, Heilongjiang, China; ^2^ College of Basic Medical Science, Heilongjiang University of Chinese Medicine, Harbin, Heilongjiang, China; ^3^ Key Laboratory of Basic and Application Research of Beiyao, Heilongjiang University of Chinese Medicine, Ministry of Education, Harbin, Heilongjiang, China

**Keywords:** *Carthamus tinctorius* L., flavonoids, pharmacology, hydroxysafflor yellow A, ethnopharmacology

## Abstract

**Background:**

*Carthamus tinctorius* L. has a long history of ethnomedicinal use for various ailments. This review focuses on the botany, ethnopharmacology, phytochemistry, pharmacological effects, and clinical applications of safflower, aiming to enhance current research in this field.

**Methods:**

The study incorporated relevant scientific literature up to April 2025. It involved the collection of both Chinese and English studies on safflower from various databases, including PubMed, Elsevier, Web of Science, Springer, ScienceDirect, Wiley, ACS, and CNKI (China National Knowledge Infrastructure). Additionally, doctoral and master’s dissertations were included in the analysis.

**Results:**

From 1978 to April 2025, various active metabolites were identified, primarily comprising flavonoids, polyacetylenes, and alkaloids, with flavonoids being the predominant group. Extracts and metabolites derived from safflower have demonstrated a range of bioactivities, including antioxidant, hepatoprotective, anti-inflammatory, and anticancer effects. In clinical practice, the effective components of safflower have been utilized in the treatment of cardiovascular and cerebrovascular diseases, diabetes, hepatobiliary conditions, poor blood circulation, sudden deafness, and other ailments.

**Conclusion:**

This review elucidates the research surrounding safflower in the domains of ethnopharmacology, phytochemistry, pharmacological activity, and clinical applications. Safflower is known to contain a diverse array of compounds, with flavonoids in particular demonstrating significant pharmacological activity. These compounds are extensively utilized in the pharmaceutical, food, and cosmetic industries, positioning safflower as a promising candidate for development and application in the treatment of various diseases. Nonetheless, research on safflower remains limited, and many active metabolites have yet to be thoroughly investigated in terms of their phytochemical and pharmacological properties. To date, only a handful of active metabolites have been isolated and assessed for their biological activity, and there is a notable deficiency in research regarding their mechanisms of action. Therefore, comprehensive studies are imperative to enhance our understanding of safflower and to substantiate its therapeutic potential.

## 1 Introduction

*Carthamus tinctorius* L., commonly known as safflower, is an effective herbal medicine with a long history of use. Its cultivation is primarily concentrated in China, India, and Western European countries (Hamdan, 2024). Safflower is associated with the liver and heart meridians and is effective in alleviating pain, promoting blood circulation, and removing blood stasis (Wang L. et al., 2023). The plant contains a variety of chemical metabolites, predominantly flavonoids, alkaloids, polyacetylenes, and polysaccharides. As medical research advances, the clinical applications of safflower in specialized fields, such as gynecology and dermatology, have become increasingly prevalent and demonstrate significant therapeutic effects (Ren et al., 2023). This review provides a comprehensive synopsis and analysis of the botany, ethnomedicine, phytochemistry, pharmacology, and therapeutic uses of safflower. Additionally, we address the limitations of previous studies and propose future research directions. Our aim is to provide a thorough analysis of safflower to assess its potential as a therapeutic agent and to recommend future research pathways that will support its ongoing development and application.

## 2 Botany

**FIGURE 1:**
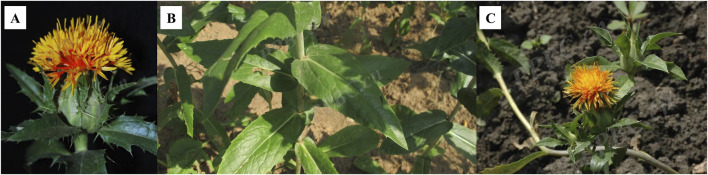
Plant flower **(A)**, leaves **(B)**, and aerial part **(C)** (http://ppbc.iplant.cn/).

**FIGURE 2:**
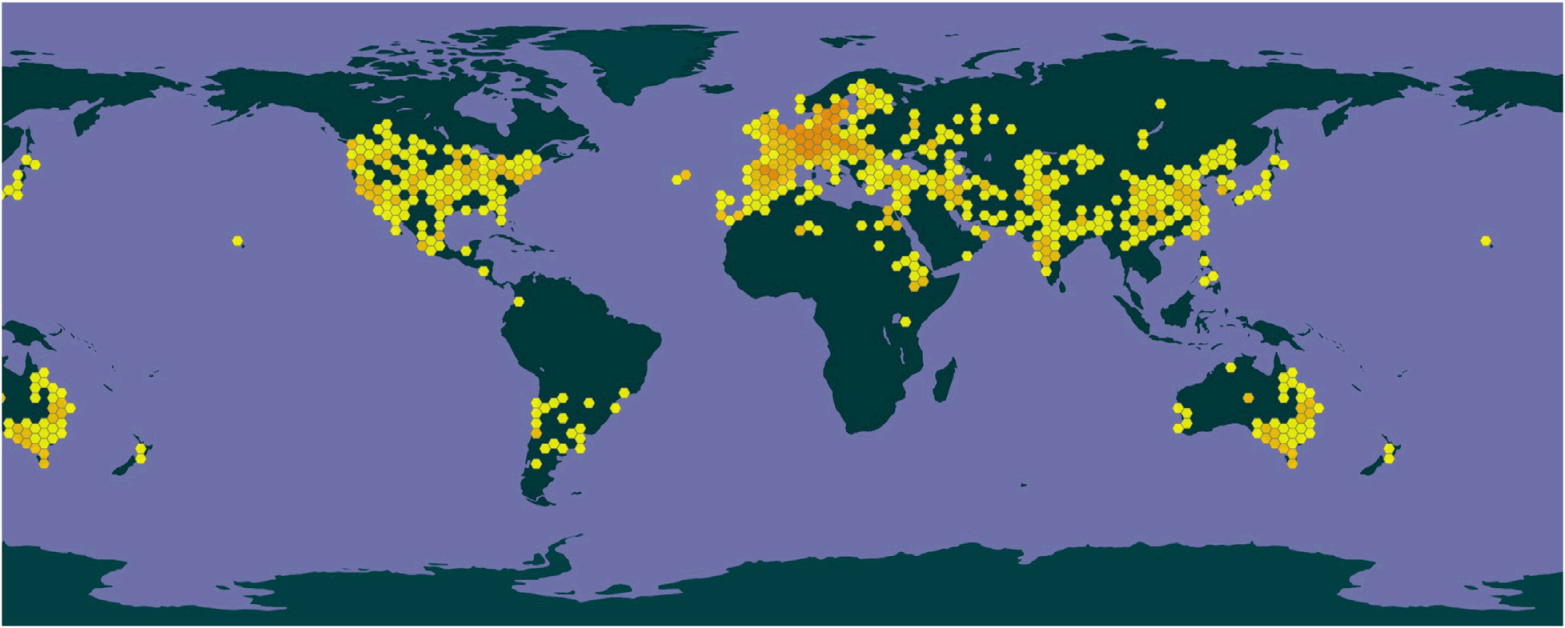
The geographical distribution of safflower.

Safflower is highly adaptable and exhibits resistance to salt, drought, and cold conditions, making it widely cultivated across China. Fragments of the stigma, corolla, and filament are commonly observed, along with elongated tubular secretory cells that can reach diameters of up to 66 μm, with secretions varying in color from yellow-brown to reddish-brown, often located near the duct (Waki et al., 2021). The outer walls of the epidermal cells at the tips of the corolla lobes display a brief, tomentose extension (Wang et al., 2015). Prominent or slightly obtuse single-celled hairs with conical apexes emerge from both the stigma and the upper epidermal cells of the style. Pollen grains possess three germination pores and tooth-like protrusions on their outer walls, measure up to 60 μm in diameter, and are ellipsoidal, olive-shaped, or orbicular (Lu et al., 2025). Calcium oxalate crystals are found within the thin-walled cells and range in size from 2 μm to 6 μm. A depiction of safflower is presented in Figure 1. The genus *Carthamus* comprises approximately 85 species, primarily distributed in India, Spain, and Sweden, with one species found in China, specifically in Xinjiang and Yunnan provinces. The geographical distribution of safflower worldwide was obtained from the GBIF online database (www.gbif.org, shown in Figure 2).

## 3 Materials and methods

### 3.1 Identification and selection of studies

The initial phase of our analysis involved systematically assessing all studies identified through keyword searches about *Carthamus tinctorius* L. Following the removal of duplicate entries, we conducted a preliminary review of titles and abstracts to evaluate their relevance based on the established inclusion criteria. A detailed examination of studies that satisfied these criteria was performed, encompassing a thorough analysis of the full text and an in-depth review of the reference lists to ensure a comprehensive understanding of the relevant literature.

### 3.2 Search strategy

We identified the studies independently using the following keywords: “*Carthamus tinctorius* L.,” “*Carthamus tinctorius*,” and “Safflower.” In addition, reported pharmacological activities and phytochemical compositions were searched as keywords. This study only includes results found before April 2025. The search was carried out in the electronic bibliographic databases, including PubMed, Elsevier, Web of Science, Springer, ScienceDirect, Wiley, ACS, and CNKI (China National Knowledge Infrastructure).

### 3.3 Inclusion and exclusion criteria

Our inclusion criteria encompassed all experimental studies investigating various aspects of *Carthamus tinctorius* L., including its botany, phytochemistry, ethnopharmacology, pharmacology, and clinical applications. Additionally, we incorporated Chinese doctoral and master’s dissertations and theses that detailed the properties of safflower. Editorials, conference abstracts, duplicate articles, review articles, and conference proceedings were excluded. Further exclusions were articles unrelated to the topic.

### 3.4 Others

ChemDraw 19.0 was used to redraw the chemical compounds. The PubChem database (https://pubchem.ncbi.nlm.nih.gov) was used to confirm the chemical classifications and structures. The whole procedure was conducted in accordance with the PRISMA statement (https://www.prisma-statement.org/).

## 4 Ethnopharmacology

The introduction of safflower to China dates back over 2,100 years. The earliest recorded application of safflower in China can be traced to the Han Dynasty, during which it was introduced from the western regions primarily as a dye. Its initial medicinal use occurred in the Eastern Han Dynasty, as documented in Zhongjing Zhang’s “Synopsis of the Golden Chamber” (《金匮要略》A.D.219). The “Natural History” (《博物志》A.D.232) notes that “it was born in the Liang and Han dynasties and the Western region and is now also grown in the Wei dynasty,” indicating that safflower was cultivated in Henan Province by at least as early as the Western Jin Dynasty. In Bao Cui’s “Ancient and Modern Annotations” (《古今注》A.D.278) from the Western Jin Dynasty, it is recorded that *Artocarpus tonkinensis* A. Chev. ex Gagnep., which has leaves resembling thistle and flowers akin to *Taraxacum mongolicum* Hand.-Mazz., originated from the West, and the locals referred to it as “Yan Zhi” in Chinese. During the Northern Wei Dynasty, “Qi Min Yao Shu” (《齐民要术》A.D.533) documented the method for planting red safflower, stating, “The flower land needs to be well-ripened. Planting occurs in late February or early March. The flowers should be picked on cool days. Picking will be exhaustive, and a method for killing flowers to make rouge is also recorded.” This indicates that mature safflower cultivation techniques were established in China by the Northern Wei Dynasty. The term “safflower” first appeared in the Song Dynasty’s “Herbal Atlas,” (《本草图经》A.D.1061) which noted, “Now it is found everywhere. People plant it in gardens, sowing seeds in the ripe ground during winter for spring seedlings.” In the Ming Dynasty, Xiangjin Wang’s “Botanical Treatise” (《群芳谱》A.D.1621) recorded that “seeds are collected in May, pounded and decocted, mixed with vinegar, and combined with vegetables for consumption. It can also be used as car fat and for making candles.” Shizhen Li also recorded in the “Compendium of Materia Medica” (《本草纲目》A.D.1590) that “the seeds of safflower can be planted in February, August, and December after rainfall, similar to the method of planting hemp. The young leaves and seedlings are also edible, and the leaves resemble those of small thistle.” This indicates that safflower has been widely utilized throughout history for various purposes, including medicinal applications, dyes, culinary uses, and oil production (Zhou et al., 2014).

From a historical perspective, safflower seeds, packets, and garlands of florets were commonly found alongside mummies in ancient Egypt (Weiss, 1971). Additionally, safflower is consumed raw in various regions of Iran (Mohamadpour et al., 2012). Safflower dye has been utilized in Italian, French, and British cuisine for both flavoring and coloring purposes. The florets have been applied in diverse ways, serving as a dye, coloring agent, flavoring, rouge, potion, and unguent (Delshad et al., 2018). The significance of safflower dyes is particularly evident in the carpet-weaving industries of Eastern Europe, the Middle East, and the Indian subcontinent (Dajue and Mündel, 1996). This specific application is reflected in the latter part of the binomial name, where plants or their derivatives are often designated with the term “tinctorius,” indicating their association with dyes (Guarrera, 2006). In Thailand, the aqueous extract of safflower flowers is widely used as a hair color promoter (Boonyaprapas and Chokchaijareonporn, 1996). In traditional Indian medicine, safflower is commonly employed for treating scabies, arthritis, and mastalgia.

This plant species is frequently used in the treatment of amenorrhea, gastric tumors, and wounds, whether of internal or external origin, according to Chinese folklore. Notably, Iranian traditional medicine recognizes safflower for treating skin patches, baldness, phlegm, and colic (Imami et al., 2010). The traditional applications of safflower in Persian medicine are documented in traditional Persian texts. The flower and seeds of safflower exhibit laxative effects, while its seed oil is utilized for conditions such as rheumatism and paralysis (Razi, 2000). Additionally, safflower facilitates the absorption of therapeutic agents by target tissues and promotes tissue contraction. It is also employed in the treatment of vitiligo, hyperpigmentation, psoriasis, oral ulcers, and for analgesic purposes.

The fruit and leaves of safflower are known to alleviate phlegm, serve as an antidote for scorpion stings, and address numbness in the limbs (Ibn, 2007). The seeds of safflower possess laxative properties and are believed to mitigate melancholic tendencies and enhance semen quality (Jorjani, 2012; Uosefi, 1999). Safflower has been utilized in Persian folk medicine for treating diabetes, phlegmatic fever, melancholia, and dropsy (Aghili Khorasani and Makhzan al-Adwiyyah, 2011). Additionally, various plants from the Compositae family are traditionally used as agents promoting abortion. The water extract of safflower is applied for painful menstruation as a sedative, serves as a laxative for constipation, and acts as an anti-inflammatory remedy in traditional medicine (Zargari, 1992). The dried floret of *Carthamus tinctorius* L., known as *Carthami flos*, has gained significant popularity due to its extensive applications in the treatment of coronary heart disease, angina pectoris, gynecological conditions, stroke, and hypertension (Chen et al., 2025).

## 5 Phytochemistry

The tubular flowers of safflower, which comprise a variety of chemical substances, are the primary sites of concentration of its active metabolites. The most prevalent of these include flavonoids, alkaloids, sterols, lignans, spermidine, alkyl diols, and polysaccharides. In addition to its tubular blossoms, the achenes are rich in unsaturated fatty acids, such as oleic acid and linoleic acid (Chen et al., 2023). The tocopherols and unsaturated fatty acids present in the seeds prevent the “three highs” (hypertension, hyperglycemia, and hyperlipidemia) and possess anti-aging properties (Wang et al., 2021).

### 5.1 Flavonoids

**FIGURE 3:**
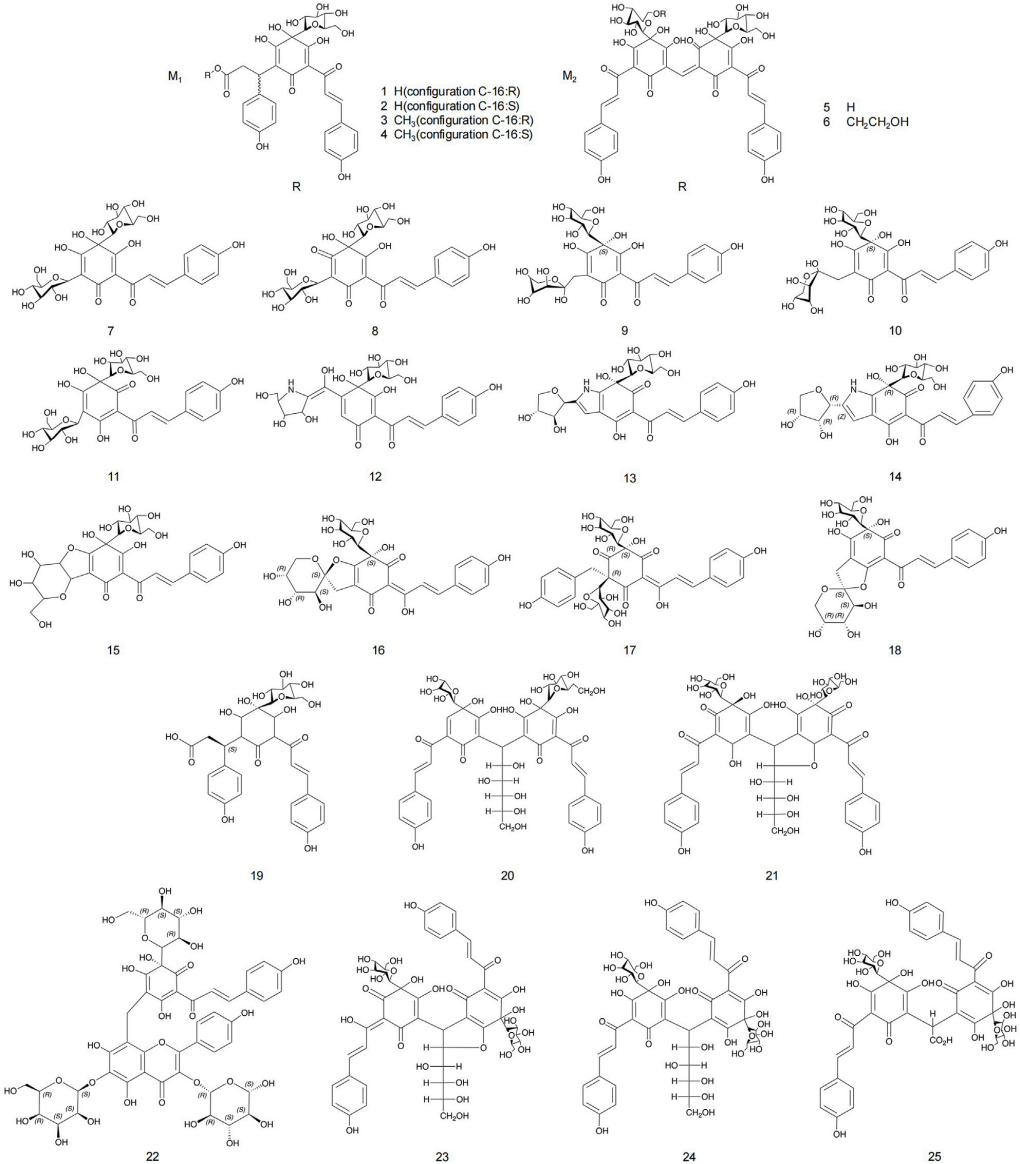
Structures of quinone chalcone, and glycoside metabolites in safflower.

**FIGURE 4:**
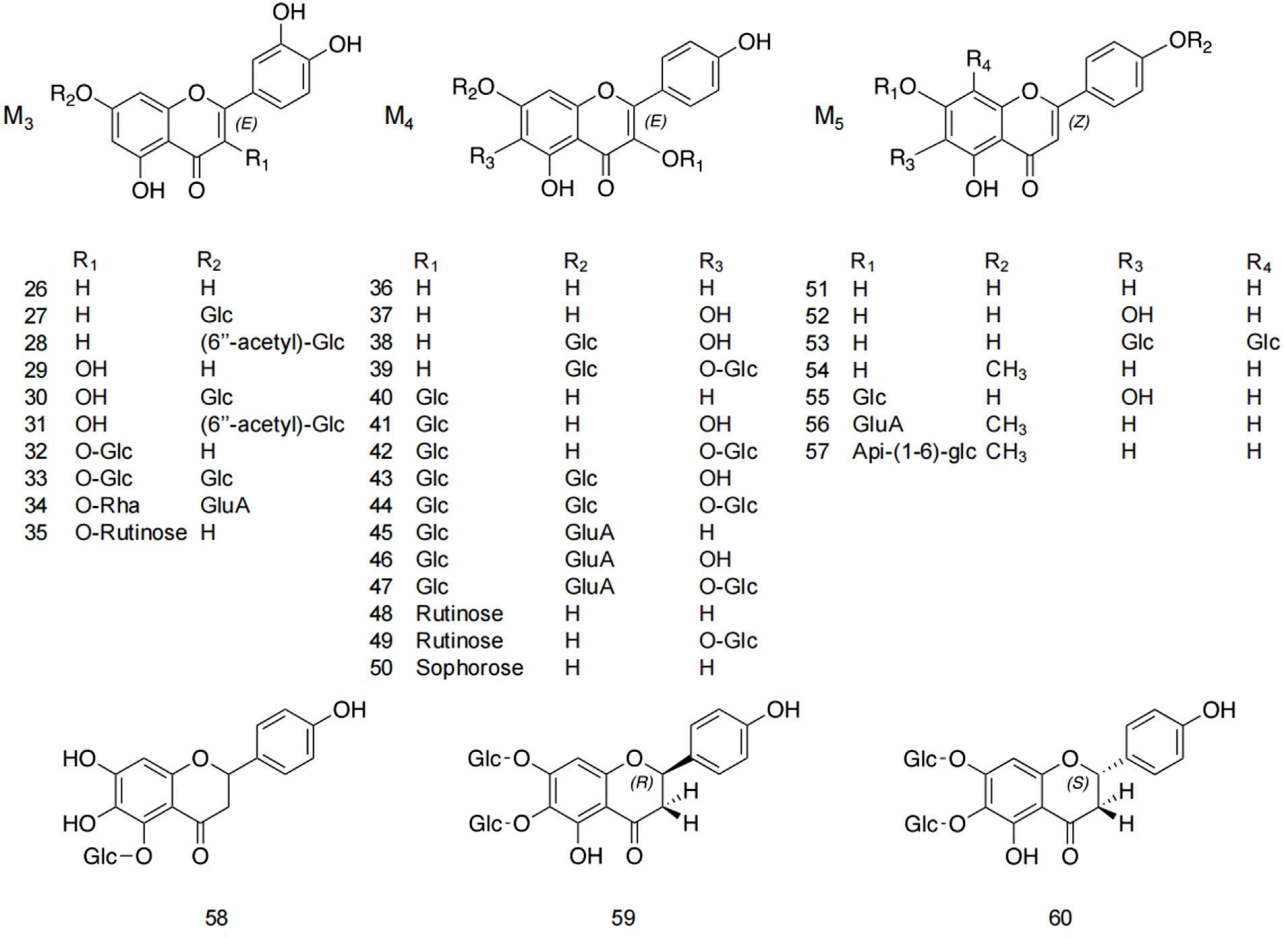
Structures of other flavonoid metabolites in safflower.

Flavonoids and flavonoid glycosides represent the most significant active metabolites in safflower and have been extensively studied within safflower research due to their close association with the pharmacological effects of this plant. The active flavonoid metabolites, known as quinone chalcone carbohydrates, encompass nearly all the safflower yellow (SY) and safflower red (SR) pigments found in safflower (Zhang J. et al., 2018). To date, 25 quinone chalcone carbohydrates have been isolated from safflower, predominantly existing as monomers, while a minority are found as bimolecular polymers. Their structures are illustrated in Figure 3. In addition to quinone chalcone carbohydrates, safflower contains flavonoid metabolites such as flavonols and dihydroflavonoids, which exhibit a range of pharmacological activities. Among these, flavonol glycosides are the most extensively studied metabolites, some of which demonstrate conformational relationships, with antioxidant activity being linked to the structure of the substituted glucose (Lee et al., 2002). Currently, 35 flavonoid metabolites have been extracted from safflower, with the primary flavonols in this category being kaempferol, apigenin, quercetin, and other derivatives. Figure 4 illustrates their specific architectures, while Table 1 provides a comprehensive list of the specific flavonoid metabolites.

**TABLE 1:** Detailed information about flavonoid metabolites in safflower.

No.	Name	Parent nucleus	Substitution	Extraction solvent	Parts used	Identification methods	References
1	Safflomin C	M_1_	R = H (Configuration: C-16:R)	Ethanol	Leaves	1D NMR; ESIMS	Cho et al. (2011)
2	Isosafflomin C	M_1_	R = H (Configuration: C-16:S)	Ethanol	Leaves Fruit	1D NMR; ESIMS	Cho et al. (2011)
3	Methylsafflomin C	M_1_	R = CH_3_ (Configuration: C-16:R)	Ethanol	Leaves Fruit	1D NMR; ESIMS	Zhao et al. (2009)
4	Methylisosafflomin C	M_1_	R = CH_3_ (Configuration: C-16:S)	Ethanol	Leaves Fruit	1D NMR; ESIMS	Zhao et al. (2009)
5	Carthamin	M_2_	R = H	Methanol	Leaves	1D NMR; ESIMS HPLC	Sato et al. (2003)
6	Hydroxyethyl ether of carthamin	M_2_	R = CH_2_CH_2_OH	Methanol	Leaves	1D NMR; ESIMS	Sato et al. (2003)
7	Hydroxysafflor yellow A			Ethanol	Leaves	1D NMR; ESIMS	Sato et al. (2003)
8	Safflomin A			Methanol	Leaves	1D NMR; ESIMS	Yue et al. (2014)
9	Hydroxysafflor yellow C			Methanol	Leaves	1D NMR; ESIMS	Yue et al. (2014)
10	Hydroxysafflor yellow B			Methanol	Leaves	1D NMR; ESIMS	Zhou et al. (2006)
11	Saffloquinoside D			Acetone	Leaves	1D NMR; ESIMS	Zhou et al. (2006)
12	Tinctormine			Chloroform	Leaves	GC-MS	Meselhy et al. (1992)
13	Cartormine			Acetone	Leaves	GC-MS	Zhou et al. (2006)
14	Isocartormin			Acetone	Leaves	GC-MS	Li et al. (2010)
15	Safflor yellow A			Chloroform	Leaves	GC-MS	Jiang et al. (2008)
16	Saffloquinoside A			Ethyl acetate	Leaves	GC-MS	Jiang et al. (2008)
17	Saffloquinoside B			Ethyl acetate	Leaves	1D NMR; ESIMS	Jiang et al. (2008)
18	Saffloquinoside C			Ethyl acetate	Leaves	1D NMR; ESIMS	Jiang et al. (2013)
19	Saffloquinoside E			Ethyl acetate	Leaves	1D NMR; ESIMS	Jiang et al. (2013)
20	Safflomin B			Acetone	Aerial parts	1D NMR; ESIMS	Sato et al. (2005)
21	Carthorquinoside B			Methanol	Leaves	HPLC	Yue et al. (2016)
22	Carthorquinoside A			Methanol	Aerial parts	1D NMR; ESIMS	Yue et al. (2016)
23	Anhydrosafflor yellow B			Methanol	Leaves	HPLC	Qu et al. (2016)
24	Safflor yellow B			Ethanol	Leaves	HPLC	Yue et al. (2014)
25	Precarthamin			Ethanol	Leaves	HPLC	Wang et al. (2006)
26	Luteolin	M_3_	R_1_ = H, R_2_ = H	Ethanol	Aerial parts	HPLC	Lee et al. (2002)
27	Luteolin-7-O-β-D-glucopyranoside	M_3_	R_1_ = H, R_2_ = Glc	Dichloromethane	Fruit	1D, 2D NMR	Lee et al. (2002)
28	Luteolin-7-O-(6″-O-acetyl)-β-D-glucopyranoside	M_3_	R_1_ = H, R_2_=(6″-acetyl)-Glc	Dichloromethane	Fruit	1D NMR	Lee et al. (2002)
29	Quercetin	M_3_	R_1_ = OH, R_2_ = H	Methanol	Leaves	1D NMR	Lee et al. (2002)
30	Quercetin-7-O-β-D-glucoside	M_3_	R_1_ = OH, R_2_ = Glc	Ethanol	Seed	GC-MS	Hattori et al. (1992)
31	Quercetin -7-O-(6″-O-acetyl)-β-D-glucopyranoside	M_3_	R_1_ = OH, R_2_=(6″-acetyl)-Glc	Ethanol	Seed	1D, 2D NMR	Lee et al. (2002)
32	Quercetin-3-O-β-D-glucoside	M_3_	R_1_ = O-Glc, R_2_ = H	Ethanol	Seed	1D, 2D NMR	Lee et al. (2002)
33	Quercetin-3, 7-di-O-β-D-glucoside	M_3_	R_1_ = O-Glc, R_2_ = Glc	Ethanol	Seed	1D, 2D NMR	Hattori et al. (1992)
34	Quercetin-3-O-α-L-rhamnoside-7-O-β-D-glucoside	M_3_	R_1_ = O-Rha, R_2_ = GluA	Ethanol	Seed	1D, 2D NMR	Hu et al. (2013)
35	Quercetin -3-O-β-rutinoside	M_3_	R_1_ = O-Rutinose, R_2_ = H	Ethanol	Seed	1D NMR; ESIMS	Tursun (2022)
36	Kaempferol	M_4_	R_1_ = H, R_2_ = H, R_3_ = H	Ethanol	Leaves	GC-MS	Lee et al. (2002)
37	6-Hydroxykaempferol	M_4_	R_1_ = H, R_2_ = H, R_3_ = OH	Ethanol	Leaves	1D NMR; HREIMS	Hattori et al. (1992)
38	6-Hydroxykaempferol-7-O-β-D-glucoside	M_4_	R_1_ = H, R_2_ = Glc, R_3_ = OH	Acetone	Aerial parts	1D,2D NMR; HREIMS	Li et al. (2017)
39	6-Hydroxykaempferol-6, 7-di-O-β-D-glucoside	M_4_	R_1_ = H, R_2_ = Glc, R_3_ = O-Glc	Acetone	Aerial parts	1D NMR; ESIMS	Li et al. (2017)
40	Kaempferol-3-O-β-D-glucoside	M_4_	R_1_ = Glc, R_2_ = H, R_3_ = H	Acetone	Root	1D NMR; ESIMS	Liu et al. (2018)
41	6-Hydroxykaempferol-3-O-β-D-glucoside	M_4_	R_1_ = Glc, R_2_ = H, R_3_ = OH	Acetone	Root	1D NMR; ESIMS	Li et al. (2017)
42	6-Hydroxykaempferol-3, 6-di-O-β-D-glucoside	M_4_	R_1_ = Glc, R_2_ = H, R_3_ = O-Glc	Acetone	Root	1D NMR; ESIMS	Hattori et al. (1992)
43	6-Hydroxykaempferol-3, 7-di-O-β-D-glucoside	M_4_	R_1_ = Glc, R_2_ = Glc, R_3_ = OH	Acetone	Root	1D NMR; ESIMS	Hattori et al. (1992)
44	6-Hydroxykaempferol-3, 6, 7-tri-O-β-D-glucoside	M_4_	R_1_ = Glc, R_2_ = Glc, R_3_ = O-Glc	Acetone	Root	1D NMR; ESIMS	Hattori et al. (1992)
45	Kaempferol-3-O-β-D-glucoside-7-O-β-D-glucoside	M_4_	R_1_ = Glc, R_2_ = GluA, R_3_ = H	Methanol	Root	1D NMR; ESIMS	Liu et al. (2018)
46	6-Hydroxykaempferol -3-O-β-D-glucoside-7-O-β-D-glucoside	M_4_	R_1_ = Glc, R_2_ = GluA, R_3_ = OH	Methanol	Leaves	1D NMR; ESIMS	Xie et al. (2015)
47	6-Hydroxykaempferol-3, 6-di-O-β-D-glucoside-7-O-β-D-glucuronide	M_4_	R_1_ = Glc, R_2_ = GluA, R_3_ = O-Glc	Methanol	Leaves	1D NMR; ESIMS	Liu et al. (2018)
48	Kaempferol-3-O-β-rutinoside	M_4_	R_1_ = Rutinose, R_2_ = H, R_3_ = H	Methanol	Leaves	1D NMR; ESIMS	Liu et al. (2018)
49	6-Hydroxykaempferol-3-O-β-D-rutinoside-6-O-β-D-glucoside	M_4_	R_1_ = Rutinose, R_2_ = H, R_3_ = O-Glc	Methanol	Leaves	1D NMR; ESIMS	Liu et al. (2018)
50	Kaempferol-3-O-β-sophorose	M_4_	R_1_ = Sophorose, R_2_ = H, R_3_ = H	Methanol	Seed	1D NMR; ESIMS	Sato et al. (2005)
51	Apigenin	M_5_	R_1_ = H, R_2_ = H, R_3_ = H, R_4_ = H	Ethanol	Seed	GC-MS	Hattori et al. (1992)
52	6-Apigenin	M_5_	R_1_ = H, R_2_ = H, R_3_ = OH, R_4_ = H	Ethanol	Seed	HPLC	Zhou et al. (2006)
53	Apigenin-6, 8-di-C-β-D-glucopyranoside	M_5_	R_1_ = H, R_2_ = H, R_3_ = Glc, R_4_ = Glc	Ethanol	Root	1D,2D NMR; HREIMS	Lee et al. (2002)
54	Acacetin	M_5_	R_1_ = H, R_2_ = CH_3_, R_3_ = H, R_4_ = H	Ethanol	Fruit	HPLC	Hattori et al. (1992)
55	Baicalin	M_5_	R_1_ = Glc, R_2_ = H, R_3_ = OH, R_4_ = H	Ethanol	Leaves	GC-MS	Hattori et al. (1992)
56	Acacetin-7-O-β-D-glucuronide	M_5_	R_1_ = GluA, R_2_ = CH_3_, R_3_ = H, R_4_ = H	Dichloromethane	Leaves	GC-MS	Lee et al. (2002)
57	5, 7-Dihydroxy-4′-methoxyflavone-7-O-β-D-apiofuranosyl-(1-6)-O-β-D-glucoside	M_5_	R_1_ = Api-(1-6)-glc, R_2_ = CH_3_, R_3_ = H, R_4_ = H	Dichloromethane	Seed	1D,2D NMR; HREIMS	Ahmed et al. (2000)
58	5, 6, 7, 4′-Rahydroxyflavanone-5-O-β-D-glucoside, neocarthamin			Chloroform	Seed	1D NMR; HREIMS	Zhou et al. (2006)
59	(2R)-4′, 5-Dihydroxyl-6, 7-di-O-β-D-glucopyranosylflavanone			Chloroform	Seed	1D NMR; HREIMS	Zhou et al. (2006)
60	(2S)-4′, 5-Dihydroxyl-6, 7-di-O-β-D-glucopyranosylflavanone			Chloroform	Seed	1D,2D NMR; HREIMS	Jiang et al. (2013)

### 5.2 Polyalkynes

**FIGURE 5:**
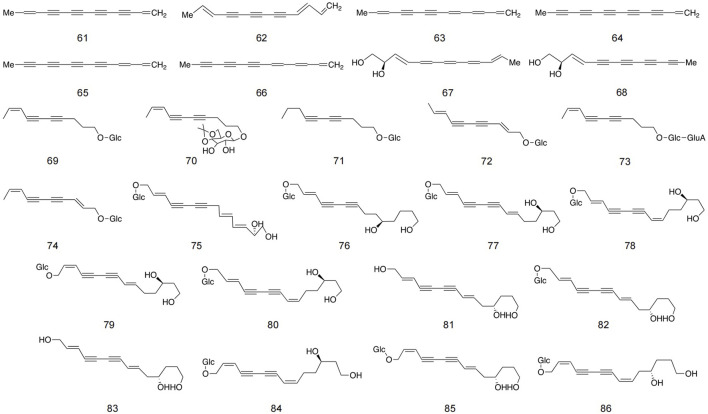
Structures of polyalkyne metabolites in safflower.

Polyalkyne metabolites in safflower are typically based on ten- and thirteen-carbon structures. The majority of glycosides in polyynes exist in an oily form, which readily aggregates in the air and is naturally unstable. However, once glycosides are formed, they transition into a powder state, thereby enhancing stability (Li et al., 2017). These metabolites is primarily located in stems, roots, blooms, and immature seeds infected by Epidermophyton (Zheng et al., 2019). Currently, 26 distinct polyynes have been isolated from safflower, with detailed structures and information provided in Figure 5 and Table 2.

**TABLE 2:** Detailed information about polyalkyne metabolites in safflower.

No.	Name	Extraction solvent	Parts used	Identification methods	References
61	1, 11-Tridecadiene-3, 5, 7, 9-tetrayne	Acetone	Root	1D NMR; ESIMS	Zheng et al. (2019)
62	1, 3, 11-Tridecatriene-5, 7, 9-triyne	Acetone	Root	1D NMR; ESIMS	Zheng et al. (2019)
63	1, 3, 5, 11-Tridecatertracene-7, 9-diyne	Acetone	Root	1D NMR; ESIMS	Zheng et al. (2019)
64	1-Tridecene-3, 5, 7, 9, 11-pentayne	Acetone	Root	1D, 2D NMR; HREIMS	Zheng et al. (2019)
65	1, 3-Tridecadiene-5, 7, 9, 11-tetrayne	Acetone	Root	1D, 2D NMR; HREIMS	Zheng et al. (2019)
66	1, 3, 5-Tridecatriene-7, 9, 11-triyne	Acetone	Root	1D, 2D NMR; HREIMS	Zheng et al. (2019)
67	Trans-3-Traiene-5, 7, 9, 11-tetraacety-1, 2-diol	Hexane	Aerial parts	1D NMR; ESIMS	Yue et al. (2014)
68	Trans, trans −3, 11-Traiene-5, 7, 9-triacety-1, 2-diol	Hexane	Aerial parts	1D NMR; ESIMS	Yue et al. (2014)
69	(8Z)- Decaene-4, 6-diyne-1-O-β-D-glucopyranoside	Petroleum ether	Aerial parts	1D NMR; ESIMS	Zhou et al. (2006)
70	4′, 6′-Acetonide-8Z-decaene-4, 6-diyne-1-O-β-D-glucopyranoside	Hexane	Aerial parts	1D NMR; ESIMS	Zhou et al. (2006)
71	4, 6-Decadiyne-1-O-β-D-glucopyranoside	Petroleum ether	Aerial parts	1D NMR; ESIMS	Zhou et al. (2006)
72	(8E)- Decaene-4, 6-diyne-1-O-β-D-glucopyranoside	Petroleum ether	Aerial parts	1D NMR; ESIMS	Li et al. (2010)
73	(8Z)- Decaene-4, 6-diyne-1-ol-1-O-β-D-glucuronyl-(1″-2′)-β-D-glucopyranoside	Petroleum ether	Aerial parts	1D, 2D NMR; HREIMS	Li et al. (2010)
74	(2E, 8Z)- Decadiene-4, 6-diyne-1-ol-1-O-β-D-glucopyranoside	Petroleum ether	Seed	1D, 2D NMR; HREIMS	Li et al. (2010)
75	(2E, 8E, 10E)- Tridecatriene-4, 6-diyne 1, 12, 13-triol-1-O-β-D-glucopyranoside	Petroleum ether	Seed	1D, 2D NMR; HREIMS	Li et al. (2010)
76	(2E)- Tetradecaene-4, 6-diyne-1, 10, 14-triol-1-O-β-D-glucopyranoside	Petroleum ether	Seed	1D, 2D NMR; HREIMS	Li et al. (2010)
77	(2E, 8E)- Tetradecadiene-4, 6-diyne-1, 12, 14-triol-1-O-β-D-glucopyranoside	Petroleum ether	Seed	1D, 2D NMR; HREIMS	Li et al. (2010)
78	(2Z, 8Z)- Tetradecadiene-4, 6-diyne-1, 12, 14-triol-1-O-β-D-glucopyranoside	Petroleum ether	Seed	1D, 2D NMR; HREIMS	Li et al. (2010)
79	(2Z, 8E)- Tetradecadiene-4, 6-diyne-1, 12, 14-triol-1-O-β-D-glucopyranoside	Petroleum ether	Seed	1D, 2D NMR; HREIMS	Li et al. (2010)
80	(2E, 8Z)- Tetradecadiene-4, 6-diyne-1, 12, 14-triol-1-O-β-D-glucopyranoside	Petroleum ether	Seed	1D, 2D NMR; HREIMS	Li et al. (2010)
81	(2E, 8E)- Tetradecadiene-4, 6-diyne-1, 11, 14-triol	Petroleum ether	Seed	1D, 2D NMR; HREIMS	Li et al. (2010)
82	(2E, 8E)-11S-Teteradecadiene-4, 6-diyne-1, 11, 14-triol-1-O-β-D-glucopyranoside	Petroleum ether	Root	1D NMR; ESIMS HPLC	Hao et al. (2010)
83	(2E, 8E)-11S-Teteradecadiene-4, 6-diyne-1, 11, 14-triol	Petroleum ether	Root	1D NMR; ESIMS HPLC	Hao et al. (2010)
84	(2Z, 8Z)-11S-Teteradecadiene-4, 6-diyne-1, 11, 14-triol-1-O-β-D-glucopyranoside	Petroleum ether	Root	1D NMR; ESIMS HPLC	Hao et al. (2010)
85	(2Z, 8E)-11S-Teteradecadiene-4, 6-diyne-1, 11, 14-Triol-1-O-β-D-glucopyranoside	Petroleum ether	Root	1D NMR; ESIMS HPLC	Hao et al. (2010)
86	(2E, 8Z)-11S-Teteradecadiene-4, 6-diyne-1, 11, 14-triol-l-O-β-D-glucopyranoside	Petroleum ether	Root	1D NMR; ESIMS HPLC	Hao et al. (2010)

### 5.3 Alkaloids and spermidines

**FIGURE 6:**
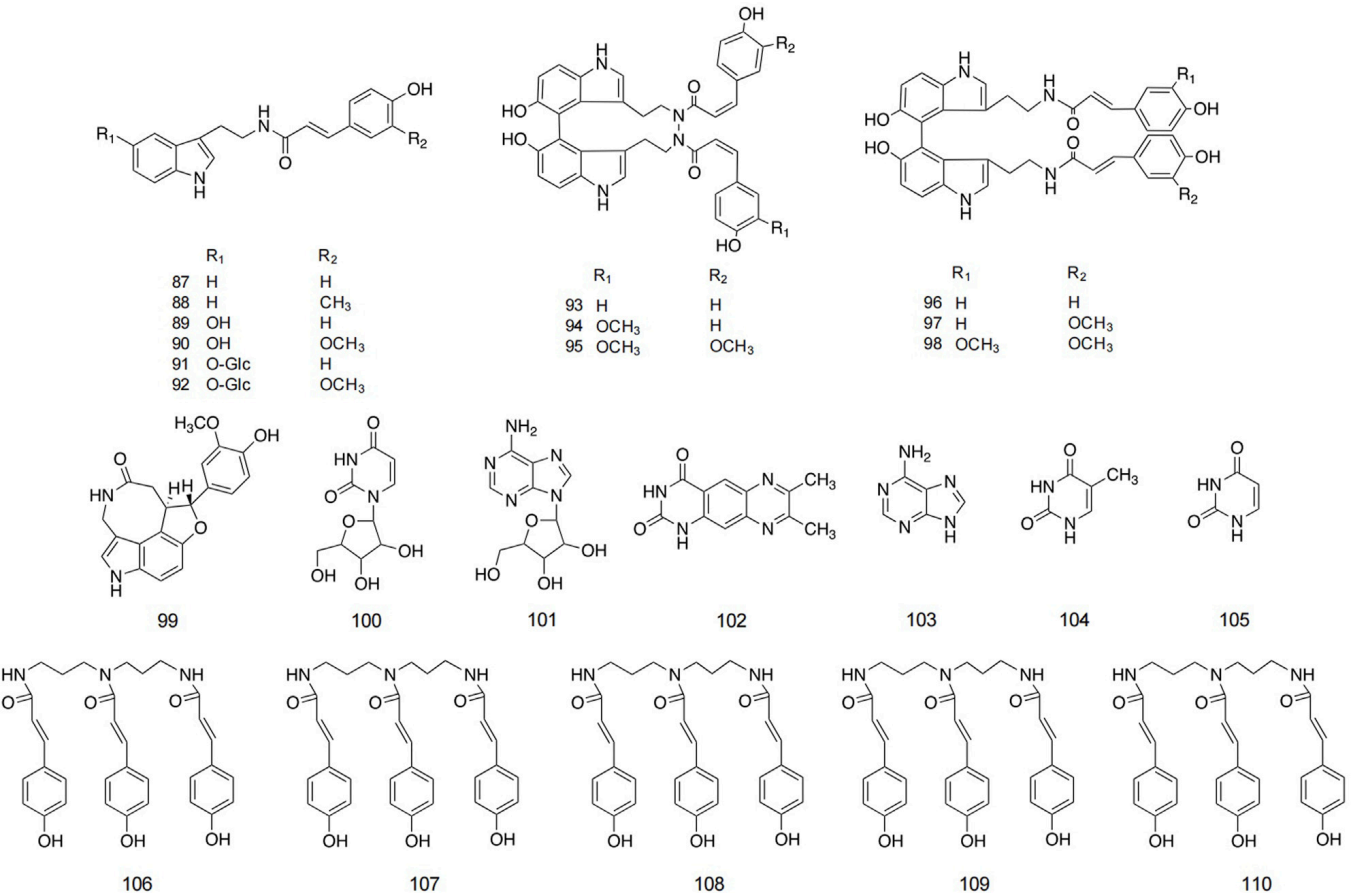
Structures of alkaloid and spermidine metabolites in safflower.

The alkaloid metabolites isolated from safflower are primarily derivatives of 5-hydroxytryptamine (5-HT), characterized by their lower polarity, and are predominantly found in safflower seeds. Additionally, a total of 13 and 11 alkaloids have been isolated from safflower oil and the dried flowers of safflower, respectively (Hao et al., 2010; Sakamura et al., 1980). Spermidine metabolites in safflower are spermidine derivatives with three coumaryl groups. Researchers successfully isolated five spermidine compounds from safflower by high-speed countercurrent chromatography (Jiang et al., 2014). Studies reported the preparation method for total spermine in safflower residue. The optimization method is to conduct three heating reflux extractions using 35 times the absolute volume of methanol, with each reflux extraction lasting for 2 h. The total extraction rate of four spermine compounds in safflower residue was 2.894 ± 0.011 mg/g (Zhao et al., 2014). Detailed structures and additional information are presented in Figure 6 and Table 3.

**TABLE 3:** Detailed information about alkaloid and spermidine metabolites in safflower.

No.	Name	Parent nucleus	Substitution	Extraction solvent	Parts used	Identification methods	References
87	N-(p-Coumaroyl)tryptamine	M_6_	R_1_ = H, R_2_ = H	Chloroform	Seed	1D NMR; ESIMS	Hao et al. (2010)
88	N-Feruloyltryptamine	M_6_	R_1_ = H, R_2_ = OCH_3_	Chloroform	Seed	1D NMR; ESIMS	Hao et al. (2010)
89	N-[2-(5-Hydroxy-1H-indol-3-yl)ethyl]-p-coumaramide	M_6_	R_1_ = OH, R_2_ = H	Ethyl acetate	Seed	1D NMR; ESIMS	Hao et al. (2010)
90	N-[2-(5-Hydroxy-1H-indol-3-yl)ethyl]-ferulamide	M_6_	R_1_ = OH, R_2_ = OCH_3_	Ethyl acetate	Seed	1D NMR; ESIMS	Hao et al. (2010)
91	N, N′-[2, 2′-(5, 5′-Dihydroxy-4, 4 ′-bi-1H-3, 3′-yl)ethyl]-di-p-coumaramide	M_6_	R_1_ = O-Glc, R_2_ = H	Ethyl acetate	Seed	1D NMR; ESIMS	Sakamura et al. (1980)
92	N-[2-[3′-[2-(p-Coumaramide)ethyl]-5, 5′-dihydroxy-4, 4′-bi-1H-indol-3-yl]-ethyl] ferulamide	M_6_	R_1_ = O-Glc, R_2_ = OCH_3_	Ethyl acetate	Seed	1D NMR; ESIMS	Zhang J. et al. (2018a)
93	N, N′- [2, 2′-(5, 5′- Dihydroxy-4, 4 ′-bi-1H-indol-3, 3 ′-yl)diethyi]-diferulamide	M_7_	R_1_ = H, R_2_ = H	Ethyl acetate	Seed	1D NMR; ESIMS	Hu et al. (2013)
94	N-[2-[5-(β-D-Glucosyloxy)-1H-indol-3-yl]ethyl]-p-coumaramide	M_7_	R_1_ = OCH_3_, R_2_ = H	Ethyl acetate	Seed	1D, 2D NMR; HRFABMS	Hu et al. (2013)
95	N-[2-[5-(β-D-Glucosyloxy)-1H-indol-3-yl]ethyl] ferulamide	M_7_	R_1_ = OCH_3_, R_2_ = OCH_3_	Ethyl acetate	Seed	1D, 2D NMR; HRFABMS	Hu et al. (2013)
96	4, 4″-bis(N-p-Coumaroyl)serotonin	M_8_	R_1_ = H, R_2_ = H	Petroleum ether	Seed	1D, 2D NMR; ESIMS	Zhou et al. (2006)
97	4-[N-(p-Coumaroyl)serotonin-4″-yl]-N-feruloylserotonin	M_8_	R_1_ = H, R_2_ = OCH_3_	Petroleum ether	Seed	1D, 2D NMR; ESIMS	Zhou et al. (2006)
98	4, 4″-bis(N-p-Feruloy)-5-serotonin	M_8_	R_1_ = OCH_3_, R_2_ = OCH_3_	Petroleum ether	Seed	1D, 2D NMR; ESIMS	Zhou et al. (2006)
99	Serotobenine			Acetone	Seed	GC-MS	Zhang J. et al. (2018a)
100	Uridine			Acetone	Aerial parts	GC-MS	Zhou et al. (2006)
101	Adenosine			Acetone	Aerial parts	GC-MS	Zhou et al. (2006)
102	7, 8-Dimethyl pyrazino [2, 3-g]quinazolin-2, 4-(1H, 3H)dione			Acetone	Seed	1D, 2D NMR; ESIMS	Zhou et al. (2006)
103	Adenine			Methanol	Seed	GC-MS	Zhou et al. (2006)
104	Thymine			Methanol	Seed	GC-MS	Zhou et al. (2006)
105	Uracil			Methanol	Seed	GC-MS	Wu et al. (2012)
106	N^1^, N^5^, N^10^-(Z)-tri-p-Coumaroylspermidine			N-butyl alcohol	Seed	1D, 2D NMR; ESIMS	Jiang et al. (2014)
107	N^1^, N^5^, N^10^-(E)-tri-p-Coumaroylspermidine			N-butyl alcohol	Seed	1D, 2D NMR; ESIMS	Jiang et al. (2014)
108	Safflospermidine A			Ethyl acetate	Seed	HPLC	Jiang et al. (2014)
109	Safflospermidine B			Ethyl acetate	Seed	HPLC	Jiang et al. (2014)
110	N^1^, N^5^-(Z)N^10^-(E)-tri-p-Coumaroylspermidine			Chloroform	Seed	1D, 2D NMR; ESIMS	Zhao et al. (2014)

### 5.4 Lignans and sterols

**FIGURE 7:**
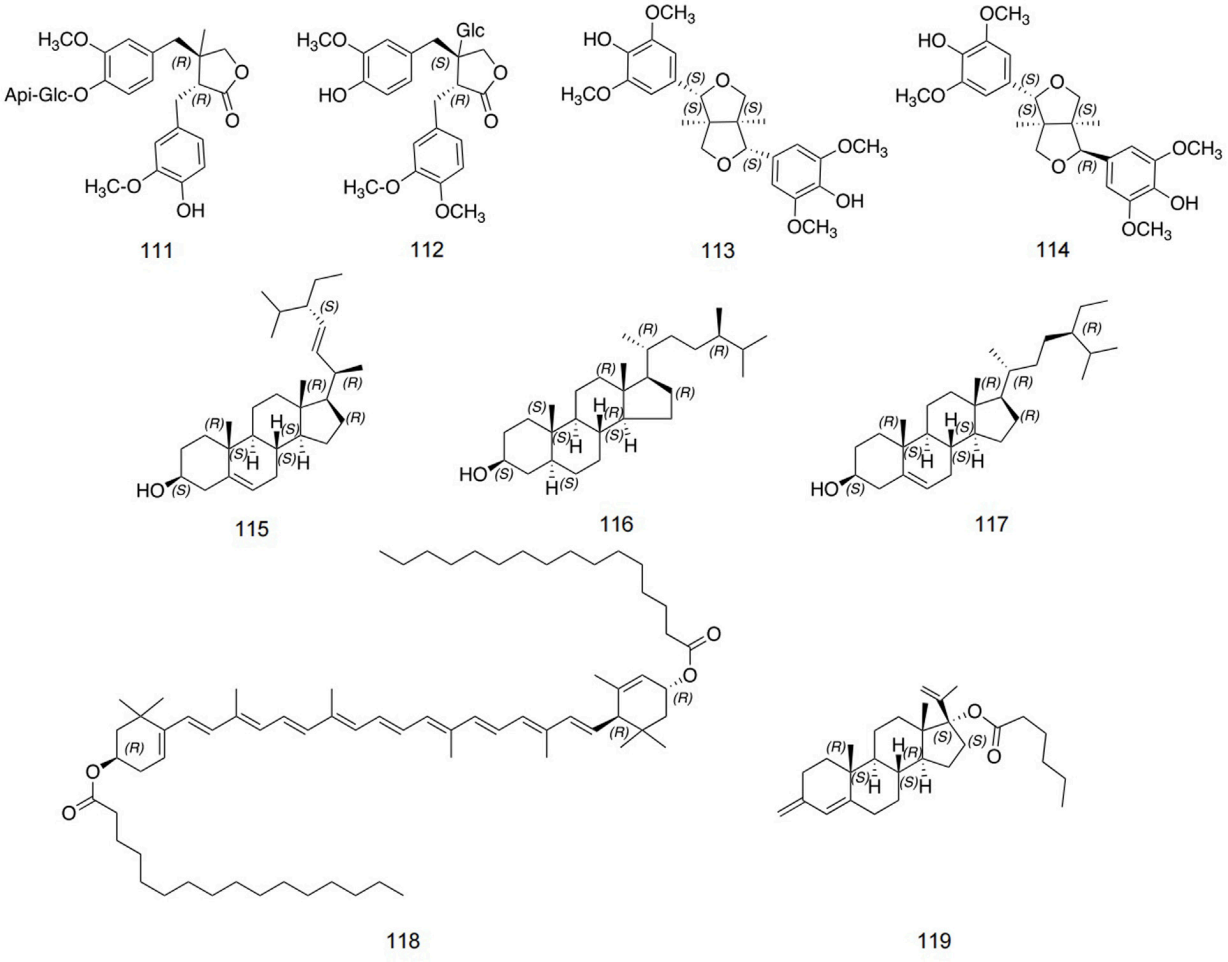
Structures of lignan and sterol metabolites in safflower.

At present, few lignans have been found in safflower, including double tetrahydrofuran syringaresinol, lirioresinol A, and so on (Peng et al., 2017). Zhou et al. identified stigmasterol, campesterol, pregnane, and so on using IR, NMR, and MS analysis methods (Zhou et al., 2014). The detailed information is shown in Figure 7 and Table 4.

**TABLE 4:** Detailed information about lignan and sterol metabolites in safflower.

No.	Name	Extraction solvent	Parts used	Identification methods	References
111	Dibenzylbutyrolactone	Ethanol	Seed	1D NMR; ESIMS	Peng et al. (2017)
112	Matairesinol-4'-O-β-D-apiofuranosyl-(1→2)-O-β-D-glucopyranoside	Acetone	Seed	1D NMR; ESIMS	Peng et al. (2017)
113	Double tetrahydrofuran syringaresinol	Acetone	Fruit	1D NMR; ESIMS	Peng et al. (2017)
114	Lirioresinol A	Acetone	Fruit	1D, 2D NMR; ESIMS	Peng et al. (2017)
115	Stigmasterol	Ethanol	Fruit	1D, 2D NMR; ESIMS	Zhou et al. (2014)
116	Campesterol	Ethanol	Seed	1D NMR; ESIMS HPLC	Zhou et al. (2014)
117	Sitosteryl-3-O-glucoside	Acetone	Seed	1D NMR; ESIMS HPLC	Zhou et al. (2014)
118	Daucosterol	Acetone	Aerial parts	1D, 2D NMR; ESIMS	Zhou et al. (2014)
119	Pregnane	Acetone	Seed	1D, 2D NMR; ESIMS	Zhou et al. (2014)

### 5.5 Other metabolites

**FIGURE 8:**
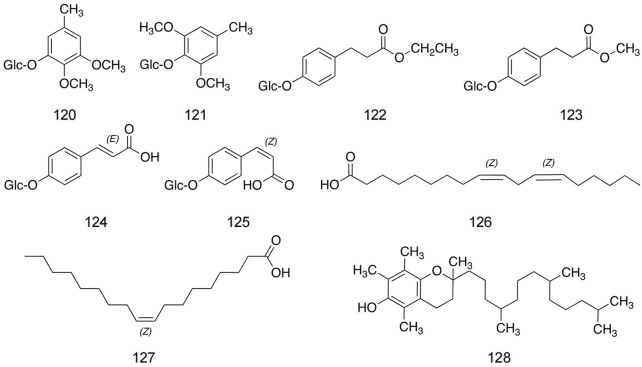
Structures of other metabolites in safflower.

Zhou et al. (2008) found three new aromatic glycosides and three known aromatic glycosides. In addition, linoleic acid, oleic acid, and tocopherol are the main components in safflower seeds. The structures and detailed information of other metabolites in safflower are shown in Figure 8 and Table 5.

**TABLE 5:** Detailed information about other metabolites in safflower.

No.	Name	Extraction solvent	Parts used	Identification methods	References
120	2, 3-Dimethoxy-5-methylphenyl-1-O-β-D-glucopyranoside	Ethyl acetate	Seed	1D, 2D NMR; ESIMS	Zhou et al. (2008)
121	2, 6-Dimethoxy-4-methylphenyl-1-O-β-D-glucopyranoside	Ethyl acetate	Seed	1D, 2D NMR; ESIMS	Zhou et al. (2008)
122	Ethyl-3-(4-O-β-D-glucopyranosyl-3-methoxyphenyl) propionate	Ethyl acetate	Seed	1D, 2D NMR; ESIMS	Zhou et al. (2008)
123	Methyl-3-(4-O-β-D-glucopyranosyl-3-methoxyphenyl) propionate	Ethyl acetate	Seed	1D, 2D NMR; ESIMS	Zhou et al. (2008)
124	Ethylsyringin	Chloroform	Leaves	1D, 2D NMR; ESIMS	Zhou et al. (2008)
125	Methylsyringin	Chloroform	Leaves	1D, 2D NMR; ESIMS	Zhou et al. (2008)
126	(9Z,12Z)-9,12-Octadecadienoic acid	Benzene	Seed	1D NMR; ESIMS HPLC	Zhou et al. (2008)
127	(Z)-9-18 (carbon) Enoic acid	Benzene	Seed	1D NMR; ESIMS HPLC	Zhou et al. (2008)
128	Tocopherol	Acetone	Seed	1D, 2D NMR; ESIMS	Zhou et al. (2008)

## 6 Pharmacological effects

**FIGURE 9:**
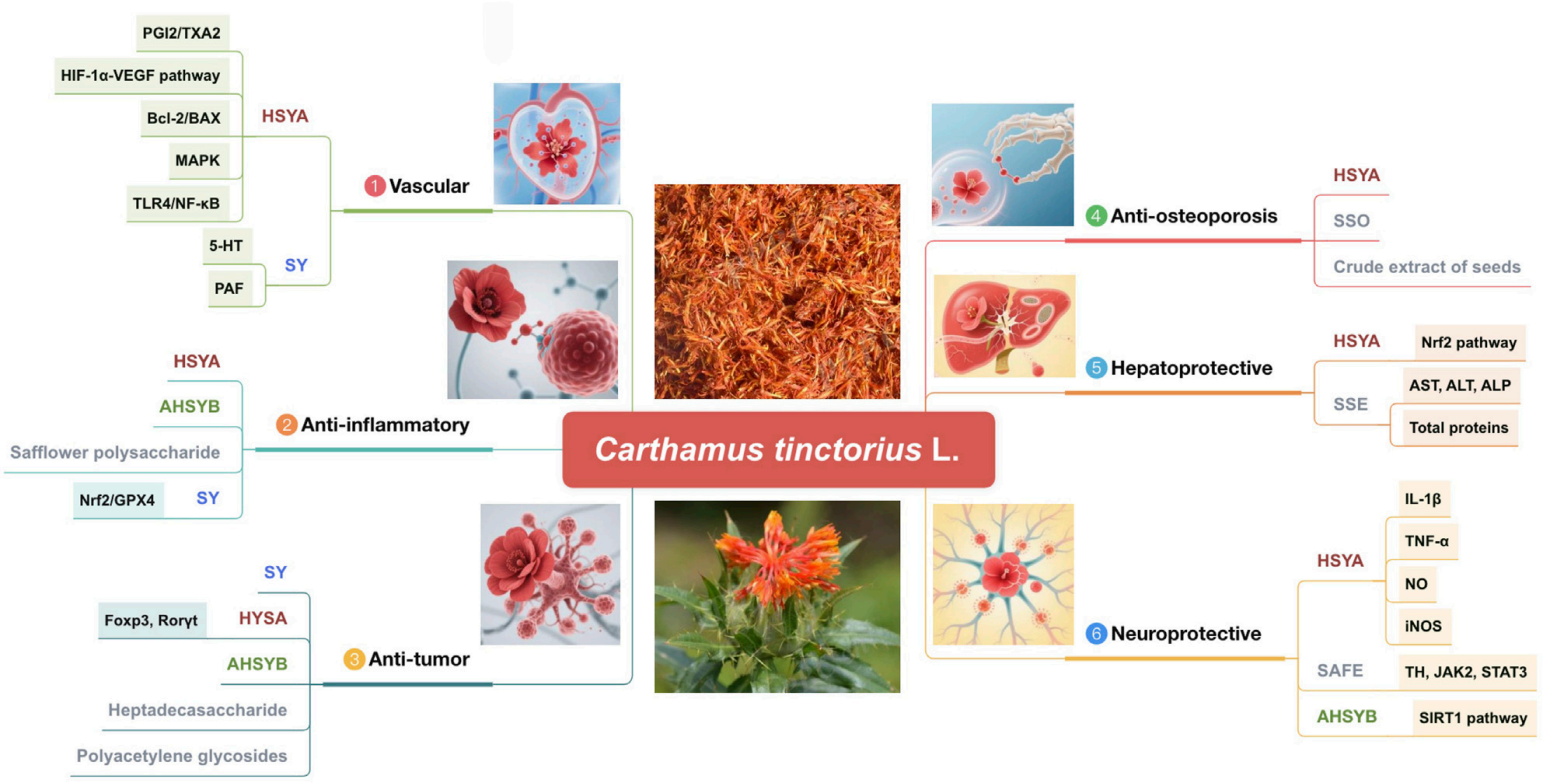
Pharmacological activities of the active metabolites of safflower (AHSYB, anhydroxysafflor yellow B; HSYA, hydroxysafflor yellow A; SAFE, safflower flavonoid extract; SSE, safflower seed extract; SSO, safflower seed oil; SY, safflower yellow).

The “Kaibao Materia Medica” (《开宝本草》A.D.973) asserts that safflower possesses the capability to activate blood circulation and promote menstruation and is primarily utilized for the treatment of menorrhagia, bruises, and injuries. According to contemporary pharmacological research, safflower has anti-inflammatory, antitumor, antioxidant, vascular, osteoporosis-preventative, and hepatoprotective properties. It also exhibits remarkable medicinal efficiency in regulating the functions of the neurological, motor, and cardiovascular systems (Kurt et al., 2025). The ensuing sections address each of these pharmacological effects. Figure 9 and Table 6 display the pharmacological properties of safflower and its active metabolites.

**TABLE 6:** Pharmacological activities of safflower metabolites.

Activities	Metabolites	Models	Doses	Negative control	Positive control	Results	References
*Vascular effects*	SY	Mice	2 mg/kg	Saline	Warfarin	Prolonging plasma prothrombin time	Zhou et al. (2014)
			2.5 mg/kg	Saline	Aspirin	Inhibiting platelet aggregation	Jiang et al. (2017)
	Safflower extract		10 g/kg	Saline	Nifedipine	Inhibiting hemodynamic alterations	Bunbupha et al. (2018)
	HSYA	Hypertensive mice	4 mg/kg	Saline	Not stated	Finding metalloproteinase expression	Wang et al. (2019)
		Mice	4 mg/kg	Saline	Icariin	Promoting bone mineralization and osteoblast viability	Liu et al. (2018)
			2 mg/kg	Saline	Clopidogrel	Reducing ADP-induced platelet aggregation	Li et al. (2017)
			Not stated	Saline	Not stated	Enhancing vascular endothelial cell viability	Yue et al. (2014)
		Human umbilical vein endothelial cells	20 μmol/L	Saline	Edaravone	Mitigating hypoxia-induced damage	Sun et al. (2013)
		Mice model of left ventricular hypertrophy	1 mg/kg	Saline	Not stated	Inhibiting cell apoptosis and metalloproteinase expression	Wang et al. (2013)
		Acute myocardial cells	10 μmol/L	Saline	Edaravone	Reducing myocardial ischemia-reperfusion injury	Ye et al. (2020)
		A model of cerebral ischemia–reperfusion injury	Not stated	Saline	Cyclosporin A	Inhibiting the opening of mitochondrial permeability transition pores	Huang et al. (2021)
		NETs-induced HUVECs and VTE mice	Not stated	Saline	Not stated	Ameliorating VTE by depleting neutrophil extracellular traps	Yan et al. (2024)
*Anti-inflammatory activities*	SY	Mice	4 mg/kg	Saline	Glutathione	Inhibiting ischemia/reperfusion injury by reducing the release of ROS	Lu et al. (2019)
	AHSYB		7.5 mg/kg	Saline	Dexamethasone	Protecting against brain I/R injury by decreasing the expression of inflammatory cytokines	Du et al. (2019)
	HSYA、SY		Not stated	Saline	Not stated	Inhibiting cardiomyocyte apoptosis after acute myocardial infarction	Zhou et al. (2014)
	HSYA	Guinea pigs	112.5 mg/kg	Saline	Dexamethasone	Enhancing the protective effect against ovalbumin-induced asthma	Guo et al. (2019)
		Mice	2 mg/kg	Saline	Minocycline	Reducing I/R-induced acute liver injury by directly attenuating macrophage activation	Jiang et al. (2017)
			3.5 mg/kg	Saline	Dexamethasone	Inhibiting the inflammatory response induced by oxygen-glucose deprivation	Li et al. (2017)
			2 mg/kg	Saline	Aspirin	A promising drug for the treatment of stroke	Sun et al. (2013)
			2 mg/kg	Saline	Dexamethasone	Reducing the loss of body weight and increasing myeloperoxidase activity	Wu et al. (2012)
			4 mg/kg	Saline	Cisplatin	Preventing the proliferation and migration of cancer cells	Zhang J. et al. (2018a)
		HUVECs/VTE mice	Not stated	Saline	Resatorvid	Inhibiting the TLR4/NF-κB pathway	Yan et al. (2024)
*Antitumor activities*	AHSYB	Mice	4 mg/kg	Saline	Cyclophosphamide	Reducing the Treg ratio in the spleen to enhance the immunity of mice	Ma et al. (2019)
		H22-bearing mice	5 mg/kg	Saline	Sunitinib	Inhibiting tumor growth by suppressing the secretion of angiogenic factors	Yang et al. (2015)
		MCF-7 cells	10 μmol/L	Saline	Not stated	Blocking the cell cycle and inducing apoptosis	Qu et al. (2019)
	Safflower polysaccharide		50 μg/mL	Saline	Not stated	Increasing in a dose- and time-dependent manner	Luo et al. (2015)
	SY	Colitis mice/Caco-2 cell models	5 μmol/L	Saline	Not stated	Inhibiting ferroptosis *via* the Nrf2/GPX4 axis	Bian et al. (2024)
	SSE	Mice	2 g/kg	Saline	Silymarin	Strong hepatoprotective and antioxidant activity	Wu et al. (2012)
	Heptadecasaccharide	Pancreatic cancer cells	10 μg/mL	Saline	Not stated	Targeting galectin-3	Hu et al. (2022)
	Polyacetylene glycosides	RAW264.7 cells	0.1 μM	Saline	Not stated	Inhibiting LPS-induced NO production	Li et al. (2021)
*Anti-osteoporosis activities*	Crude extract	MC3T3-E1 cells	20 μg/mL	Saline	Calcitonin	Preventing bone loss	Jang et al. (2007)
	SSO	Osteoporotic demodulated mice	3 g/kg	Saline	Not stated	A potential role in ameliorating osteoporosis	Alam et al. (2006)
	SSE	A preclinical single-walled model in dogs	95 g/kg	Saline	Calcitonin	Improving bone formation	Kim et al. (2002)
	HSYA	HSCs	2 μmol/L	Saline	IFN-γ	Inhibition of HSC activation and cell proliferation	Dong (2019)
*Hepatoprotective activities*	Safflower injection	Lymph retentive encephalopathy in mice	Not stated	Saline	ARBs	Treating lymph retentive encephalopathy	Pan et al. (2012)
		Mice injected with carbon tetrachloride	Not stated	Saline	Not stated	A promising anti-fibrotic agent for chronic liver disease	Zhang S. M. et al. (2018b)
	Safflower seed	Postmenopausal women	Not stated	Saline	α-tocopherol	Strong antioxidant and potential osteoprotective effects without hepatotoxicity	Cho et al. (2011)
	Safflower extract	Mice	Not stated	Saline	Not stated	Inhibiting hemodynamic alterations	Bunbupha et al. (2018)
*Neuroprotective activities*	Safflower petal extracts		12 g/kg	Saline	Not stated	Free radical scavenging and neuroprotective effects	Abuova et al. (2022)
	Safflower flavonoid extract		Not stated	Saline	L-Dopa	Significant anti-PD effects	Lei et al. (2020)
	Kaempferol-3-O-rutinoside/AHSYB	Molecular docking analysis	Not stated	Saline	Not stated	Potential drug candidate for PD prevention	Ablat et al. (2022)
	SY and HYSA	AD rat model	2 mg/kg	Saline	Betamethasone	Alleviating amyloid β1-42-induced glutamate cycle disorder	Hou et al. (2020)
	HYSA	Mice	2 mg/kg	Saline	Dexamethasone	Inhibiting the expression of NF-κB p65 and iNOS	Tiwari et al. (2018)
	HYSA and AHSYB		Not stated	Saline	Not stated	Inhibiting apoptosis and reducing oxidative stress	Fangma et al. (2021)

### 6.1 Vascular effects

Recent pharmacological studies have demonstrated that SY, the active metabolite in safflower, significantly prolongs plasma prothrombin time and activated partial thromboplastin time, reduces plasma fibrinogen content, and inhibits platelet aggregation induced by adenosine diphosphate (ADP) in rat models (Zhou et al., 2014). Additionally, SY significantly inhibits platelet aggregation induced by platelet-activating factor (PAF), 5-HT release, and increases intraplatelet free Ca^2+^ levels (Jiang et al., 2017). Hydroxysafflor yellow A (HSYA) has been shown to reduce ADP-induced platelet aggregation in a dose-dependent manner, achieving a maximum inhibition rate of 41.8%. The mechanism of action of HSYA may be attributed to its inhibition of thrombosis, reduction of platelet aggregation, and regulation of prostacyclin/prothrombin (PGI2/TXA2) as it significantly enhances blood rheological parameters such as whole blood viscosity, plasma viscosity, erythrocyte deformability, and aggregation, while having no significant effect on erythrocyte cumulative pressure (Li et al., 2017). Notably, safflower has been observed to prevent venous thrombosis but not arterial thrombosis, indicating that safflower may exhibit greater efficacy in venous thrombosis models, which are characterized by higher fibrin content in stasis-dependent thrombosis.

HSYA enhances vascular endothelial cell viability under hypoxic conditions by activating the HIF-1α-VEGF pathway and modulating the Bcl-2/Bax ratio (Yue et al., 2014). By preventing apoptosis and cell cycle arrest, HSYA may also mitigate hypoxia-induced damage to human umbilical vein endothelial cells (Sun et al., 2013). A 4-week treatment of rats with safflower extract to study its effect on renal vascular hypertension showed that safflower extract inhibited hemodynamic alterations and vascular remodeling in 2K-1C hypertensive rats and had potent antioxidant activity (Bunbupha et al., 2018). Experiments on a rat model of left ventricular hypertrophy injected with different doses of HSYA found that HSYA at doses of 20 mg/kg and 40 mg/kg could inhibit cell apoptosis and metalloproteinase expression by enhancing the ratio of Bcl-2/Bax (Wang et al., 2013). The inflammatory response is the main cause of acute myocardial cell apoptosis, and HSYA reduces myocardial ischemia–reperfusion injury by reducing autophagy and inhibiting the inflammatory response (Ye et al., 2020). Some studies have also established a model of cerebral ischemia–reperfusion injury and found that HSYA inhibits the opening of the mitochondrial permeability transition pore and limits the output of mitochondrial cytochrome C (CytC) by regulating the mitogen-activated protein kinase (MAPK) signaling pathway, thus helping to improve cerebral ischemia–reperfusion injury (Huang et al., 2021). Recent findings revealed that HSYA exerted protective effects against ferroptosis in neutrophil extracellular traps (NETs)-induced HUVECs and venous thromboembolism (VTE) mice. HSYA ameliorates VTE by depleting neutrophil extracellular traps through the inhibition of the TLR4/NF-κB pathway, thus providing a novel therapeutic strategy for treating VTE (Yan et al., 2024). Modern pharmacological research indicates that safflower can dilate blood vessels, enhance microcirculation, increase blood flow, and stimulate uterine activity. Safflower injection has emerged as a popular therapeutic option, exhibiting sedative and analgesic properties (Wan et al., 2020).

### 6.2 Anti-inflammatory activities

Numerous studies have shown that the anti-inflammatory activity of safflower may be related to its flavonoid metabolites, which exhibit strong and effective anti-inflammatory activity *in vitro* and *in vivo*. *In vitro* studies have shown that HSYA with anhydroxysafflor yellow B (AHSYB) inhibited a variety of inflammatory responses, including inhibition of PAF proliferation and asthma-related inflammatory responses in human bronchial smooth muscle cells (HBSMCs) (Guo et al., 2019). According to the research of Bacchetti et al., safflower polyphenol extract and HSYA from safflower had high antioxidant activity. They could also reduce the sensitivity of low-density lipoprotein to copper-induced lipid peroxidation and regulate the oxidative stress induced by tert-butyl hydrogen peroxide in human skin fibroblasts (HSFs), but at high concentrations, these extracts could promote oxidation (Bacchetti et al., 2020). *In vivo* studies have shown that the direct injection of HSYA (50 mg/kg, 75 mg/kg, and 112.5 mg/kg) into guinea pigs enhances the protective effect against ovalbumin (OVA)-induced asthma (Zheng et al., 1996).

Safflower extract inhibited ischemia/reperfusion (I/R) injury in rats by reducing the release of reactive oxygen species (ROS) and attenuating the inflammatory response (Lu et al., 2019). AHSYB could protect against brain I/R injury by decreasing the expression of inflammatory cytokines in rats (Du et al., 2019). HSYA and SY inhibit cardiomyocyte apoptosis after acute myocardial infarction (AMI) and protect against myocardial ischemia in rats (Zhou et al., 2014). The therapeutic effect of HSYA on liver I/R injury was tested by constructing a mouse model, and the results showed that HSYA could reduce I/R-induced acute liver injury by directly attenuating macrophage activation under inflammatory conditions (Jiang et al., 2017). When the effects of HSYA treatment on microglia ischemia were examined in a mouse model, the results showed that HSYA inhibited the inflammatory response induced by oxygen-glucose deprivation (OGD) (Li et al., 2017). HSYA was administered to rats with focal cerebral ischemia to see if it had neuroprotective effects, and the results of the study showed that HSYA is a promising drug for the treatment of stroke (Sun et al., 2013). Similarly, HSYA was injected into mice in three doses (26.7 mg/kg/day, 40 mg/kg/day, and 60 mg/kg/day). The results showed that HSYA reduced the loss of body weight, increased myeloperoxidase activity, and inhibited the inflammatory response in the lungs induced by bleomycin (Wu et al., 2013). Researchers established colitis models in mice *via* DSS and in Caco-2 cells *via* lipopolysaccharide. Further analyses revealed that SY could inhibit ferroptosis *via* the Nrf2/GPX4 axis in both *in vivo* and RSL3-induced Caco-2 cell models. Importantly, the antiferroptotic and protective effects of SY were nullified by Nrf2 knockout *in vivo* and by the use of ML385 *in vitro*. The effects of SY on ulcerative colitis (UC) are strongly associated with the Nrf2 pathway. SY might be a potential candidate for the treatment of UC, which provides an important reference for investigating the mechanisms of flavonoid compounds involved in preventing inflammatory diseases (Bian et al., 2024).

### 6.3 Antitumor activities

Safflower extracts have been shown to have a strong inhibitory effect on several types of cancer in both *in vivo* and *in vitro* tests. In one *in vivo* experiment, the anticancer effect of HSYA was investigated using a mouse model, and it was found that HSYA can effectively prevent the proliferation and migration of cancer cells and can induce apoptosis, a result that provides a scientific basis for an anticancer agent for human hepatic cell carcinoma (HCC) (Zhang S. M. et al., 2018). The proportion of FOXP3-expressing Tregs in the spleen and the expression of Foxp3 and Rorγt mRNA decreased following treatment with certain doses of HSYA. HSYA inhibited tumor growth without detrimental effects on the weight of the mice, indicating that HSYA may be suitable as a novel therapy for HCC patients (Ma et al., 2019). In an *in vitro* study, AHSYB was found to block the MCF-7 cell cycle and induce apoptosis (Qu et al., 2019). Similarly, in an experiment to observe the effect of safflower polysaccharide on the proliferation and metastasis of MCF-7 human breast cancer cells, its inhibitory effect was found to increase in a dose-time-dependent manner (Luo et al., 2015). It has also been found that HSYA has an effect on angiogenesis in H22-bearing mice, and the results suggest that HSYA could significantly inhibit tumor growth by suppressing the secretion of angiogenic factors, indicating that HSYA is a candidate for the prevention and treatment of HCC (Yang et al., 2015). All these experimental results suggest that SY analogs, as flavonoid metabolites in safflower, are related to the antitumor process of safflower. Additionally, a highly branched heptadecasaccharide can target galectin-3 and inhibit pancreatic cancer cell growth (Hu et al., 2022). The polyacetylene glycoside (5R)-5-acetoxy-8,10,12-tetradecatriyne-1-O-β-D-glucopyranoside exhibited anti-inflammatory activity by inhibiting LPS-induced NO production in RAW264.7 cells (Li et al., 2021).

### 6.4 Anti-osteoporosis activities

HSYA has the potential to prevent and treat glucocorticoid-induced intraocular pressure elevation (GCIOP) by promoting bone mineralization, osteoblast viability, and bone collagen expression and inhibiting bone resorption (Liu et al., 2018). Another study showed that a crude extract of seeds affected osteoblast differentiation and intracellular calcium ion concentration in MC3T3-E1 cells, suggesting that the crude extract of seeds has the ability to prevent osteoporosis and prevent bone loss (Jang et al., 2007). A study of the effects produced by safflower seed oil (SSO) on osteoporotic de-ovulated rats showed a potential role of SSO in ameliorating osteoporosis (Alam et al., 2006). The effect of safflower seed extract (SSE) on periodontal tissue regeneration was evaluated in a preclinical single-walled model in dogs and showed improved bone formation (Kim et al., 2002).

### 6.5 Hepatoprotective activities

Injection of HSYA into rat hepatic stellate cells (HSCs) showed inhibition of HSC activation and cell proliferation, suggesting it as a potential candidate for the prevention and treatment of liver fibrosis (Dong, 2019). The effect of HSYA on brain changes induced by lymphoretentive encephalopathy in rats, a test that supports the idea that HSYA can treat lymphoretentive encephalopathy (Pan et al., 2012). Hepatic fibrosis was significantly reduced in rats injected with carbon tetrachloride every 2 weeks (5 mg/kg) within 12 weeks, indicating that HSYA is a promising anti-fibrotic agent for chronic liver disease (Zhang S. M. et al., 2018). Additionally, Wu et al. explored a rat liver injury model induced by CCl4 through *in vivo* experiments and observed that safflower seed extract (SSE) could reduce the serum levels of AST, ALT, ALP, and total protein in model rats. Through HE staining analysis, it was determined that HSYA effectively alleviates liver injury by exerting a significant hepatoprotective effect *via* the Nrf2 pathway (Wu et al., 2013). Studies have shown that SSE tocopherol has strong hepatoprotective and antioxidant activity when administered at doses up to 2 g/kg in a rat model (Wu et al., 2013).

### 6.6 Neuroprotective activities

Parkinson’s disease (PD) and Alzheimer’s disease (AD) are neurodegenerative diseases. Safflower petal extracts have been shown to have free radical scavenging and neuroprotective effects (Abuova et al., 2022). Safflower flavonoid extract (SAFE) showed significant anti-PD effects, which might be due to the anti-inflammatory activity of flavonoids (Lei et al., 2020). Molecular docking analysis revealed that key components of SAFE, such as kaempferol-3-O-rutinoside or AHSYB, can bind to proteins such as TH, JAK2, STAT3, and α7-nAChR (Ablat et al., 2022). Thus, SAFE is a potential drug candidate for PD prevention.

SY and HYSA can protect nerves by alleviating amyloid β1-42-induced glutamate cycle disorder in an AD rat model and by improving synaptic structural plasticity, leading to enhanced learning and memory (Hou et al., 2020). In particular, HYSA can partially inhibit the expression of NF-κB p65 and iNOS and downregulate the levels of IL-1β, TNF-α, and NO, leading to the suppression of inflammatory responses, the attenuation of LPS-induced midbrain neurotoxicity and neuroinflammation, and the alleviation of LPS-induced dopaminergic neuronal damage (Tiwari et al., 2018). HYSA and AHSYB may improve cell viability, decrease neuronal apoptosis, reduce infarct volume, improve neurological function, inhibit apoptosis, and reduce oxidative stress, which suggests that HYSA and AHSYB are potential drugs for the treatment of brain ischemia/reperfusion (I/R) injury *via* the SIRT1 pathway (Fangma et al., 2021).

## 7 Clinical applications

Safflower is known for its ability to activate blood circulation, disperse stasis, and relieve pain. In Western medicine, safflower is recognized for its uterine-stimulating effects and is widely used to treat gynecological conditions such as dysmenorrhea, abdominal masses, chronic pelvic inflammatory disease, and pelvic stasis syndrome (Zhou et al., 2006). In 2013, Dong et al. summarized the medical records of patients who used SY at their institution, revealing that 59.32% of the 880 records pertained to cardiovascular issues, while 20.00% were related to cerebrovascular diseases (Dong et al., 2013). Notably, the combination of SY for injection with insulin and azithromycin yielded promising results. Concurrently, Li et al. demonstrated that blood-activating and stasis-transforming medications can exert myocardial protective effects through various pathways and targets (Li et al., 2017). Among these, safflower can decrease serum lactate dehydrogenase (LDH) levels by enhancing the body’s antioxidant enzymes, such as superoxide dismutase (SOD) and glutathione peroxidase (GSH-PX).

Ovarian vein syndrome, also referred to as pelvic stasis syndrome, is a significant cause of gynecological pelvic pain, characterized by chronic discomfort, a marked increase in fatigue, and, in severe cases, symptoms indicative of neurological depletion. In Western medicine, surgical interventions for severe cases, such as total transabdominal hysterectomy or round ligament suspension, have been widely accepted (Zhou et al., 2014). However, patients, especially women, often find these treatments to be more distressing and may struggle to accept them. Due to their pharmacological effects, safflower and safflower-containing formulations have gained popularity in clinical treatments, providing significant benefits to female patients (Zhang S. M. et al., 2018). Dysmenorrhea, a prevalent gynecological condition, is primarily attributed to the weakening of qi and blood in the uterus due to blood stasis and qi stagnation, according to TCM (Zhang et al., 2011). The clinical application of Honghua injection for stimulating the “San Yin” acupoint may benefit patients with dysmenorrhea, as it can modulate sympathetic nerve fibers and relax the pelvic floor and uterine smooth muscles. This treatment is particularly effective for primary dysmenorrhea.

Diabetes mellitus is classified as a form of “thirst” in TCM, and is often characterized by abnormalities in blood glucose and lipid levels, with an irreversible onset leading to long-term complications such as renal failure (Qu et al., 2019). In clinical practice, Liu et al. treated patients with diabetic nephropathy for several years. When assessing the effects of safflower redox on type 2 diabetic nephropathy, using metformin and glibenclamide as positive control drugs, they found that SY significantly reduced malondialdehyde (MDA) levels and increased SOD content in the body, thereby improving antioxidant capacity, lowering blood glucose levels in diabetic nephropathy patients, enhancing insulin resistance, and ultimately providing protective effects on the kidneys (Liu et al., 2018). Notably, a clinical trial conducted by Zhang et al. categorized the clinical applications of safflower and provided experimental evidence demonstrating its efficacy in reducing insulin resistance and renal oxidative stress. The study revealed that safflower inhibits the expression of growth factors in renal tubules, decreases renal interstitial fibrosis, and possesses anti-inflammatory, anti-fibrotic, anticoagulant, and antioxidant properties. Moreover, it enhances blood viscosity, coagulation, and aggregation, which collectively contribute to improved renal blood perfusion. These effects are crucial in preventing the progression of kidney disease (Zhang S. M. et al., 2018).

In TCM theory, the liver and bile are considered cognate, with bile being closely associated with the liver. Wu et al. concluded that the metabolites extracted from safflower not only inhibit bile acid synthesis and promote the excretion of bile acids and bilirubin from the liver, but also alleviate jaundice, hepatomegaly, liver injury, and liver failure caused by bile stasis. The mechanism of action is related to the farnesoid X receptor (FXR) and pregnane X receptor (PXR), which regulate bile synthesis and expression. On one hand, FXR activation downregulates cholesterol 7α-hydroxylase (CYP7A1), thereby alleviating hepatocellular injury caused by bile acid accumulation. On the other hand, it controls the expression of related proteins, reduces hepatic uptake, and promotes the metabolism of bile acids and bile salts (Wu et al., 2013). Clinical trials have shown that oral treatment with safflower seed granules in postmenopausal women over a period of time had strong antioxidant and potential osteoprotective effects without hepatotoxicity (Cho et al., 2011).

Sudden deafness is a symptom of sudden sensorineural hearing loss, which has various causes and mechanisms and has been relatively under-researched clinically. SY and *Ginkgo biloba* extract may alleviate the symptoms of sudden deafness resulting from abnormal microcirculation in the ear, edema in the inner ear canal, and nutritional damage to the ear canal. Wang conducted a study on patients with sudden deafness by measuring their hearing, blood lipids, blood rheology, neutrophils, and lymphocytes following drug administration (Wang, 2020). The study confirmed that SY improves inner ear microcirculation in patients with sudden deafness by reducing blood lipids, decreasing capillary permeability, and inhibiting inflammatory exudation. The cost of SY is lower than that of Ginkgo biloba extract, known as “gold nadol” in Chinese, and its therapeutic effect is significant.

In addition to treating physical diseases, a recent survey of 752 Saudis who had previously tried safflower for depression and anxiety showed that 279 (37.1%) reported that safflower was effective, whereas 389 (51.73%) reported some improvement (Albaiz, 2022). Consistent with the survey, a systematic review of scientific articles published between 2010 and 2020 showed that safflower flower extracts have an anxiolytic effect as effective as diazepam (Meneses et al., 2023). Due to its nutritional and health benefits, many safflower products, such as painkillers, health drinks, skin lotions, tablets, and other nutritional supplements, are currently on the market. The combination of safflower and other ingredients is effective in the treatment of some diseases. It was reported that GuHong injection, composed of safflower and the chemical drug N-acetyl-L-glutamine, has great value in clinical settings for cerebrovascular diseases, such as ischemic stroke and related diseases (Wang Q. et al., 2023). A safflower and peach kernel herb pair is widely used in TCM for the treatment of liver fibrosis (Huang et al., 2023; Yuan et al., 2023).

## 8 Conclusion and perspective

Based on data gathered from both traditional and contemporary literary sources, this article outlines the historical applications, chemical constitution, and extensive pharmacological activities of safflower. Through years of contemporary research, primary metabolites such as polyalkynes, flavonoids, alkaloids, and polysaccharides have been identified, isolated, and their pharmacological properties confirmed. Pharmacological studies have substantiated the traditional uses of safflower, particularly in the management of dysmenorrhea and urinary tract infections (Adamska and Biernacka, 2021). To fully comprehend the mechanisms of action of safflower, further in-depth research on the intricate pharmacological effects of its metabolites, along with comprehensive analyses of all phytochemicals, is necessary.

Thin-layer chromatography and microscopy techniques are the only methods authorized by the Chinese Pharmacopoeia for the identification of safflower. Therefore, it is crucial to develop a reliable, precise, and scientifically valid identification technique to ensure the authenticity of the product. The majority of ost safflower medicinal materials are derived from wild sources. Consequently, attention should be directed towards developing large-scale cultivation methodologies to preserve the sources of safflower medicinal materials and minimize confusion between products and substitutes. A unified standard system and quality grade standards should be prioritized in research (Liao et al., 2019). Alkaloids and flavonoids are recognized as the primary pharmacologically active metabolites among several bioactive compounds identified in safflower, along with newly isolated metabolites. However, basic research on the pharmacological activities of safflower remains limited, primarily concentrating on the activities of the extracted components (Pu et al., 2019). Therefore, future research should strengthen the investigation of the biological activity of other chemical metabolites, and the interactions and structure–activity relationships between alkaloids and flavonoids. Additionally, clinical studies are necessary to effectively evaluate the efficacy, adverse reactions, and toxicity of safflower.

At present, research on safflower predominantly focuses on quinones, and the variety of safflower preparations utilized in clinical practice remains limited. The separation and investigation of other chemical components are insufficient, leading to an incomplete understanding of the effective components and a lack of depth in pharmacological mechanism research. It is essential to elucidate the pharmacological mechanisms of action to better guide clinical drug use and facilitate new drug development. Increasingly, there is recognition of the significance of the prevention of chronic diseases and the challenges posed by an aging population. Safflower possesses both dietary and medicinal properties (Snoke et al., 2022). Although the range of safflower products developed is currently limited, the safflower industry is experiencing significant growth. Various enterprises have transformed certain chemical components abundant in safflower into marketable products, including safflower tea, safflower pigment, safflower vinegar, and safflower seed oil (Nasiri et al., 2021). Moving forward, it is imperative to focus on the research and development of the safflower industry to create a broader array of products that contribute to human health.

## Abbreviations

5-HT, 5-hydroxytryptamine; 5-HT, 5-hydroxytryptamine; ADP, adenosine diphosphate; AHSYB, anhydroxysafflor yellow B; AMI, acute myocardial infarction; CYP7A1, cholesterol 7α-hydroxylase; CytC, cytochrome C; FXR, farnesoid X receptor; GSH-PX, glutathione peroxidase; HCC, hepatic cell carcinoma; HSC, hepatic stellate cell; HSFs, human skin fibroblasts; HSYA, hydroxysafflor yellow A; I/R, ischemia/reperfusion; MAPK, mitogen-activated protein kinase; MDA, malondialdehyde; OGD, oxygen-glucose deprivation; OVA, ovalbumin; PAF, platelet-activating factor; PXR, pregnane X receptor; ROS, reactive oxygen species; SOD, superoxide dismutase; SR, safflower red; SSE, safflower seed extract; SSO, safflower seed oil;SY, safflower yellow; TCM, traditional Chinese medicine.
